# Supplementary material for: Serum level of HDL particles are independently associated with long-term prognosis in patients with coronary artery disease: The GENES study
Source: Sci Rep. 2020 May 18;10:8138. doi: 10.1038/s41598-020-65100-2 (PMC7234989; doi:10.1038/s41598-020-65100-2)
Supplement: Supplementary file 1 — Supplementary information. [file 41598_2020_65100_MOESM1_ESM.docx]

**Serum level of HDL particles are independently associated with long-term prognosis in patients with coronary artery disease: The GENES study**

Thibaut Duparc ^1,2^, Jean-Bernard Ruidavets ^3^, Annelise Genoux ^1,2,4^, Cécile Ingueneau^1,2,4^, Souad Najib ^1,2^, Jean Ferrieres ^3,5^, Bertrand Perret ^1,2,4^, Laurent O. Martinez ^1,2^

^1^ Institut National de la Santé et de la Recherche Médicale (INSERM), UMR 1048, Institute of Metabolic and Cardiovascular Diseases, Toulouse, France,

^2^ University of Toulouse, UMR1048, Paul Sabatier University, Toulouse, France,

^3^ Department of Epidemiology, Health Economics and Public Health, UMR1027 INSERM, Toulouse University, Toulouse University Hospital (CHU), Toulouse, France,

^4^ Service de Biochimie, Pôle biologie, Hôpital de Purpan, CHU de Toulouse, Toulouse, France,

^5^ Fédération de Cardiologie, Toulouse University Hospital, Toulouse, France.

**Supplementary material:**

|  | Full cohort  (n = 214) | Alive  (n = 136) | Dead  (n = 78) | p |
| --- | --- | --- | --- | --- |
|  |  |  |  | <0.02 |
| Myocardial infarction (MI) | 112 (52.3%) | 66 (48.5 %) | 46 (59.0 %) |  |
| Other Ischemic heart disease (IHD) | 21 (9.8%) | 9 (6.6 %) | 12 (15.4%) |  |
| Revascularization | 81 (37.9%) | 61 (44.9 %) | 20 (25.6%) |  |
| **Supplementary Table 1. Distribution of past history events** | | | | |
| Sorted in the following order: first MI even if they had revascularization then IHD then revascularization | | | | |

|  | **Included**  (n = 214) |  | **Non included**  (n = 620) |  | p ^c^ |
| --- | --- | --- | --- | --- | --- |
| Age, years | 60.3 (7.8) |  | 60.1 (8.2) |  | 0.81 |
| Smoking, pack year | 39.9 (37.7) |  | 36.9 (35.3) |  | 0.29 ^d^ |
| Smoking habits, % |  |  |  |  | 0.09 |
| Current smoking | 23.4 |  | 18.9 |  |  |
| Past smoking | 59.3 |  | 63.5 |  |  |
| Never smoking | 17.3 |  | 17.6 |  |  |
| Alcohol, g/day | 29 (30.2) |  | 28 (32.6) |  | 0.75 ^d^ |
| Physical activity, high level ^a^, % | 10.3 |  | 12.6 |  | 0.46 ^e^ |
| Hypertension ^b^, % | 65.6 |  | 69.2 |  | 0.33 ^e^ |
| Treatment Hypertension, % | 45.5 |  | 43.4 |  | 0.66 ^e^ |
| Dyslipidemia ^b^, % | 63.7 |  | 71.1 |  | 0.05 ^e^ |
| Treatment dyslipidemia, % | 57.5 |  | 65.8 |  | 0.04 ^e^ |
| Diabetes ^b^, % | 27.2 |  | 27.2 |  | 0.99 ^e^ |
| Treatment diabetes, % | 24.3 |  | 24.5 |  | 0.92 ^e^ |
| Waist circumference, cm | 99.1 (10.8) |  | 98.9 (11.0) |  | 0.82 |
| BMI, kg/m^2^ | 27.2 (3.8) |  | 27.4 (4.1) |  | 0.34 |
| Systolic blood pressure, mm Hg | 137 (20.7) |  | 140.5 (20.4) |  | 0.03 |
| Heart rate, beats/min | 64 (13.3) |  | 64.2 (11.6) |  | 0.81 |
| Triglycerides, g/L | 1.73 (0.97) |  | 1.70 (1.02) |  | 0.66 ^f^ |
| Total cholesterol, g/L | 2.05 (0.42) |  | 2.00 (0.44) |  | 0.56 |
| LDL-C, g/L | 1.29 (0.37) |  | 1.23 (0.39) |  | 0.09 |
| HDL-C, g/L | 0.43 (0.13) |  | 0.43 (0.12) |  | 0.41 |
| ApoA-I, g/L | 1.21 (0.23) |  | 1.25 (0.22) |  | 0.06 |
| Lipoprotein A-I, g/L | 0.45 (0.13) |  | 0.48 (0.15) |  | 0.06 |
| eGFR < 30 mL / min, % | 2.4 |  | 1.5 |  | 0.37 ^g^ |
| hs-CRP, mg/L | 16.7 (28.2) |  | 11.6 (19.0) |  | 0.001 ^d^ |
| NT-proBNP, pg/mL | 800 (1812) |  | 607 (1433) |  | 0.23 ^d^ |
| hs-TnT, pg/mL | 207 (490) |  | 123 (342) |  | 0.02 ^d^ |
| LVEF < 50%, % | 29 |  | 26.1 |  | 0.37 ^e^ |
| Gensini score | 46.4 (40.1) |  | 47.2 (40.5) |  | 0.89 ^f^ |
| Duration of CAD (months) | 42.4 (63.8) |  | 42.7 (66.6) |  | 0.92 ^d^ |
| **Supplementary Table 2. Comparison between CAD patients included and patients non included.**  Data are expressed in mean (SD) or % (n).  BMI, body mass index; hs-CRP, high-sensitivity C-reactive protein; eGFR, estimated glomerular filtration rate; hs-TnT, high-sensitive cardiac troponin T; NT-ProBNP, N-terminal pro-brain natriuretic peptide; LVEF, left ventricular ejection fraction; CAD, coronary artery disease.  ^a^ “high” physical activity during 20 min at least twice a week versus “low” physical activity once a week or less. ^b^ Hypertension, systolic blood pressure ≥ 140 mmHg or diastolic blood pressure ≥ 90 mmHg or treatment; Dyslipidemia, total cholesterol ≥ 2.50 g / L or treatment; Diabetes, glucose ≥ 7.8 mmol / L or treatment. ^c^ Student’s t-test, unless otherwise stated. ^d^ Wilcoxon-Mann-Withney test; ^e^ Chi-squared test. ^f^ tests performed on log transformed data. ^g^ Fischer’s exact test. | | | | | |
